# Validation of a standardized MRI method for liver fat and T2* quantification

**DOI:** 10.1371/journal.pone.0204175

**Published:** 2018-09-20

**Authors:** Chloe Hutton, Michael L. Gyngell, Matteo Milanesi, Alexandre Bagur, Michael Brady

**Affiliations:** Perspectum Diagnostics, Oxford, United Kingdom; Universita degli Studi di Pisa, ITALY

## Abstract

**Purpose:**

Several studies have demonstrated the accuracy, precision, and reproducibility of proton density fat fraction (PDFF) quantification using vendor-specific image acquisition protocols and PDFF estimation methods. The purpose of this work is to validate a confounder-corrected, cross-vendor, cross field-strength, in-house variant LMS IDEAL of the IDEAL method licensed from the University of Wisconsin, which has been developed for routine clinical use.

**Methods:**

LMS IDEAL is implemented using a combination of patented and/or published acquisition and some novel model fitting methods required to correct confounds which result from the imaging and estimation processes, including: water-fat ambiguity; T2* relaxation; multi-peak fat modelling; main field inhomogeneity; T1 and noise bias; bipolar readout gradients; and eddy currents. LMS IDEAL has been designed to use image acquisition protocols that can be installed on most MRI scanners and cloud-based image processing to provide fast, standardized clinical results. Publicly available phantom data were used to validate LMS IDEAL PDFF calculations against results from originally published IDEAL methodology. LMS PDFF and T2* measurements were also compared with an independent technique in human volunteer data (n = 179) acquired as part of the UK Biobank study.

**Results:**

We demonstrate excellent agreement of LMS IDEAL across vendors, field strengths, and over a wide range of PDFF and T2* values in the phantom study. The performance of LMS IDEAL was then assessed *in vivo* against widely accepted PDFF and T2* estimation methods (LMS Dixon and LMS T2*, respectively), demonstrating the robustness of LMS IDEAL to potential sources of error.

**Conclusion:**

The development and clinical validation of the LMS IDEAL algorithm as a chemical shift-encoded MRI method for PDFF and T2* estimation contributes towards robust, unbiased applications for quantification of hepatic steatosis and iron overload, which are key features of chronic liver disease.

## Introduction

Hepatic steatosis and iron overload are two key features of chronic liver disease [1]. Quantitative MRI can provide surrogate metrics for these features and, in some cases, predict clinical outcomes [2]. A healthy liver should contain relatively low amounts of fat, though it is estimated that up to 1 in 3 people worldwide have non-alcoholic fatty liver disease (NAFLD) [3], and this number is increasing. In the UK Biobank imaging enhancement study, for example, which aims eventually to include 100,000 nominally healthy participants [4], liver fat was measured using MRI proton density fat fraction (PDFF) in 4,949 participants (aged 45–73 years) [5]. The results showed that although the median fat level was 2.11%, a further 19.9% of participants had fat levels > 5.5% (the commonly accepted risk level for NAFLD [6]), 9.2% of the participants (n = 455) had fat levels > 10%, and 84 (1.7%) participants had fat levels > 20%. In the same cohort, liver iron levels were measured using quantitative T2*-mapping, then converted to liver iron concentration in mg Fe/g dry weight [7]. Preliminary results suggested that a higher than expected number of participants for a normal population had elevated iron levels [8]. This is important for healthcare, since iron overload significantly increases the risk of liver disease, including cirrhosis and cancer, but can be treated effectively once diagnosed. Furthermore, liver fibrosis and inflammation can be quantified using the MR T1 relaxation parameter, but only after correcting for iron level [9]. For these reasons, multiparametric MRI, combining T1, T2* and PDFF, has been proposed as a comprehensive method for the non-invasive diagnosis of liver disease, to provide early diagnosis, treatment monitoring and an alternative to the limited method of tissue biopsy [1,2]. Note that T2* measurements can also be reported in terms of the reciprocal R2* and both have their advantages. In our work, we have adopted T2* because it has dimensions of time and so is consistent with T1 measurements.

MRI-based quantification of PDFF and iron is well-established and validated. Standard techniques for measuring PDFF rely on multiecho gradient echo (GRE) imaging with chemical shift-based methods to decompose the signal from in-phase and opposed-phase images into fat and water [10,11]. For iron quantification, standard approaches measure T2 relaxation times using spin-echo imaging [12] or T2* relaxation times [13,14] using multiecho GRE imaging. T2* relaxometry has the advantage over T2 relaxometry of shorter acquisition times, so reduced physiological artefacts, such as those resulting from breathing and motion. When multiecho GRE data are acquired using in-phase and opposed-phase images, they can be used to derive both T2* and PDFF maps. However, it has been established that for higher levels of iron, increased T2* decay yields errors in PDFF estimates; similarly, T2* decay is influenced by higher fat levels, leading to errors in T2* estimates, hence iron quantification [15,16].

Such confounds are addressed by the IDEAL approach (Iterative Decomposition of water and fat with Echo Asymmetry and Least-squares estimation), by simultaneously estimating fat and T2* decay [17,18], to provide iron-corrected PDFF estimates fat-corrected iron quantification [19]. The IDEAL technique also embodies use of a more realistic fat spectrum model with multiple resonant frequencies, enabling more accurate PDFF and T2*. It also facilitates shorter and more closely-spaced echo times, which is important for reducing motion artefacts and imaging higher iron levels.

Various studies have demonstrated the accuracy, precision and reproducibility of PDFF quantification using methods based on the IDEAL approach [20–22]. However, to date, much of the published work has used vendor-specific versions of the image acquisition protocol and PDFF estimation methods (e.g. IDEAL IQ, GE Healthcare or mDIXON Quant, Philips Healthcare). Although in some cases encouraging results were reported across vendors and field strength, the differences that have been observed have been attributed to differences in the acquisition and estimation techniques. For example, in a recent study [23], the agreement of PDFF measurements were determined among readers, two MR scanner vendors, and two field strengths. PDFF estimation was performed using two different vendor products with the precise data acquisition protocol and PDFF calculation being specific to each vendor. Although the results were reported to be highly reproducible across readers, field strengths, and imaging platforms, a number of small but systematic and significant differences in PDFF were observed between scanner platforms. These were attributed to differences in the pulse sequences and PDFF calculation. This highlights the need for a liver fat and T2* quantification method that is standardized across field strength and MR scanner vendor, not least to: facilitate longitudinal assessment of individuals; characterize disease across larger populations; and to normalize clinical decision making. The overall goal of this work is to both develop and validate such a vendor neutral, field strength independent (“standardised”) method for liver fat and T2* quantification.

To this end, we licensed and acknowledge the intellectual property for the ‘IDEAL’ method from the University of Wisconsin [17], which we then implemented in software in-house. In this paper, we distinguish between the published concept and licensed intellectual property and the software that we have developed to implement it, and we refer to our software as “*LMS (LiverMultiScan) IDEAL*”. *LMS IDEAL* incorporates some of the methods reported in the referenced publications and a number of novel algorithmic steps, which we have found necessary to address different confounds resulting from the imaging process. *LMS IDEAL* has been designed to use image acquisition protocols that can be installed on most MRI scanners and cloud-based image processing to provide fast, standardized clinical results, which are consistent for data acquired on different vendors’ scanners and at different field strengths. We present two studies to validate *LMS IDEAL*. First, PDFF values were calculated for publicly available phantom data and compared with independently calculated and published values using the same phantom data, which had previously shown excellent results for reproducibility across sites, vendors, field strengths and image acquisition protocols [24]. Second, *LMS IDEAL* PDFF and T2* values were calculated in human volunteer data acquired as part of the UK Biobank data study, and the results were compared with the PDFF and T2* values estimated in the same participants using a different, but widely-used “standard” MR imaging protocol and quantification method.

## Methods

### Calculation of PDFF and T2*

**Fig 1:**
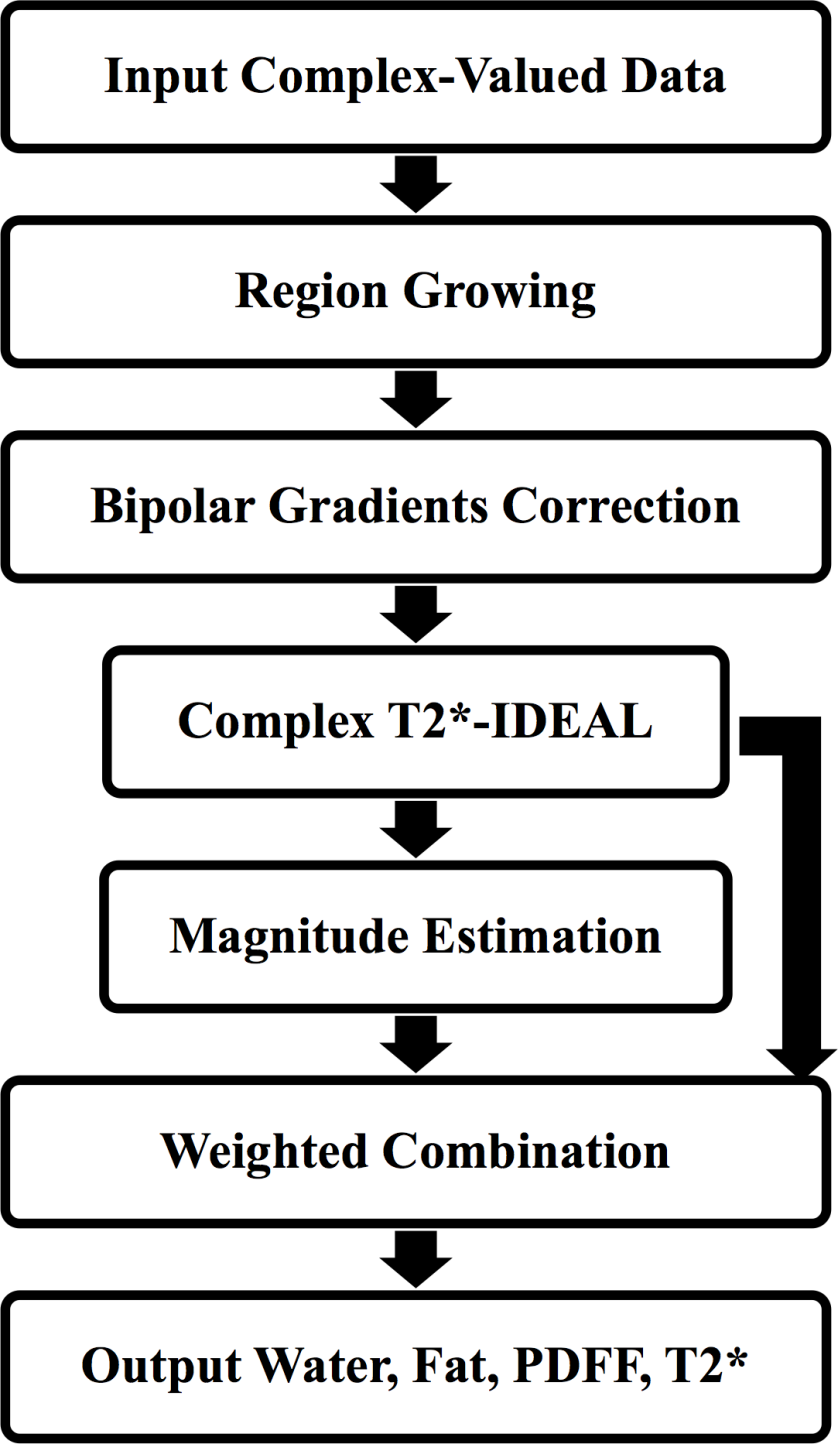
Flowchart showing LMS IDEAL.

As noted above, the ‘IDEAL’ method, from the University of Wisconsin [17,18] was implemented in-house (LMS IDEAL), to perform simultaneous water and fat decomposition and estimation of T2* decay in the presence of field (B_0_) inhomogeneity (see Fig 1 for flowchart). The most basic version of the IDEAL method [25] fits a signal equation describing the complex-valued data *s*_*n*_ at each pixel resulting from multi-echo spoiled gradient echo (SPGR) images acquired at each echo time *t*_*n*_, as

$$s_{n}=\left(\sum_{j=1}^{M}\rho_{j}\;e^{i2\pi {{∆f}_{j}t}_{n}}\right){.e}^{i2\pi \psi t_{n}}$$ (Eq 1)

where: *ρ*_*j*_ is the intensity of the *j*th of *M* chemical species, with chemical shift (in Hz) of Δ*f*_*j*_ with respect to water, and *ψ* is the local value of the “field map” (Hz). The model fitting proposed in the patent and published papers first uses an iterative least squares estimation method to determine *ψ*, which is then demodulated from the original signal, and decomposed into estimates of the two chemical species, water and fat. PDFF values are then calculated by dividing the estimates for fat by the estimate of fat plus water. *LMS IDEAL* extends the basic version of the IDEAL method, which estimates a “complex field map” (replacing *ψ* with $\hat{\psi }$ in Eq 1) and decomposes it into field map *ψ* and T2* (or 1/R2*) from the real and imaginary parts of $\hat{\psi }$ respectively [18],

$$\hat{\psi }=\psi +i/(2\pi \;T2*)$$ (Eq 2)

Given a poor starting estimate, large deviations in the B_0_ field, and/or low SNR, estimation of the B_0_ field may converge to the wrong solution. In such a case, the assignment of signal to water and to fat can be ‘swapped’, resulting in ‘fat/water swaps’ either in individual pixels or, more usually, in larger contiguous regions. To mitigate this, *LMS IDEAL* includes the method based on [26], which initially down-samples the complex echoes then uses a region growing algorithm to encourage B_0_ estimates to be similar among neighbouring pixels. This (pseudo-regularisation) step provides an initial estimate of the B_0_ field map in a central region of the image where it is most likely to be reliable, so that the final estimated field map is less likely to contain artefactual discontinuities across the image and therefore reduces ambiguities in fat and water estimation.

*LMS IDEAL* also incorporates a model that assumes that the spectrum of fat has several prominent peaks [15,16]. The model can be adjusted to approximate the spectrum for liver fat, which is required for human imaging, or for peanut oil, which is typically used in fat phantoms. For consistency with the published data, the (human) liver fat model comprises six peaks with frequency shifts relative to the water peak (ppm) of 0.6, -0.5, -1.95, -2.60, -3.4 and -3.8 and relative amplitudes of 0.047, 0.039, 0.006, 0.12, 0.7 and 0.088 [27]. Conversely, the peanut oil phantom model also comprises six fat peaks which are corrected for room temperature by adjusting the frequency shifts relative to water by 0.1ppm (this assumes 22°C), resulting in frequency shifts (ppm) of 0.5, -0.49, -2.04, -2.7, -3.5, and -3.9, and relative amplitudes of 0.048, 0.039, 0.004, 0.128, 0.694 and 0.087 [24,27].

Performing IDEAL model fitting to the complex-valued data results in estimates of fat and water which cover the entire range of possible PDFF values (from 0% to 100%). However, the accuracy of such estimates depends on the consistency of phase information across the acquired echoes. Since LMS IDEAL was implemented to be used with data acquired on different vendors’ scanners, the accuracy of the phase data can not always be guaranteed. The negative impact of inconsistent phase information can be removed by discarding the phase information from the data and estimating the IDEAL model using just the magnitude of the data (as described in [28]). It has been reported that model fitting to magnitude only information restricts estimates of PDFF values to the the range [0%, 50%], which can result in ambiguity between fat and water estimates. For this reason, based on [28], *LMS IDEAL* uses the initial, full PDFF range estimates of fat and water resulting from the complex-valued data estimation steps (including the region growing to avoid discontinuities in the field map) as starting estimates for model fitting using the magnitude of the data. The two sets of water and fat estimates (from the complex and magnitude model fitting) are then combined so that values closer to 50% are weighted towards the complex estimates and values closer to 0% and 100% are weighted towards the magnitude results [28]. Furthermore, to address the effect of a positive noise bias for low PDFF values, as a result of the magnitude data estimation steps, *LMS IDEAL* includes the approach described in [29], where, rather than trying to estimate the fat parameter as an independent signal component from the water, the model is adjusted to estimate the combined fat plus water signal, and the estimated water parameter is subtracted from the result.

Finally, the *LMS IDEAL* complex-valued data estimation steps were implemented to allow for bipolar readout gradients, as well as the more typically used monopolar readout gradients. For bipolar readout gradients, inconsistent phase information will have an opposite effect on odd and even echoes resulting in spatially varying estimates of water and fat across the image. To address this, model fitting in *LMS IDEAL* is modified by additionally including a signal modulation, which is equal and opposite in consecutive echoes (as described in [30]). This enables a pixelwise phase error map to be estimated, to which a single linear function can be fitted across the image to correct for the effect of the bipolar readout gradient.

The *LMS IDEAL* version used in this study was implemented in Matlab (The MathWorks, Inc.) with executable mex-files, consisting of compiled C++ routines using ITK libraries (www.itk.org).

### PDFF in publicly available phantom data

We downloaded 28 sets of publicly-available phantom data (http://dx.doi.org/10.5281/zenodo.48266) [24], which were acquired using one phantom at six sites, covering: 3 vendors (GE Healthcare, Siemens and Philips); 2 field strengths (1.5T and 3T); and 2 protocols. One of the six sites had two sets of data (one at the beginning of the phantom study and one at the end), to give (6+1)x2x2 = 28 sets of data in total. The phantom consisted of 11 vials with oil/water concentrations: 0%, 2.6%, 5.3%, 7.9%, 10.5%, 15.7%, 20.9%, 31.2%, 41.3%, 51.4%, 100%. The data from each system, and for each protocol, involved 6 echoes of complex-valued multi-echo gradient echo MR images, as well as the PDFF map calculated by the authors and used to report published PDFF values, (referred to here as the Reference PDFF map).

We used our in-house implementation of *LMS IDEAL*, with a 6-peak peanut oil fat model corrected for room temperature (see previous section), to calculate *LMS IDEAL* PDFF maps for the 28 sets of phantom data, which each consisted of 3 slices. Circular regions of interest (ROIs) of approximately 19.5mm diameter were placed manually on each vial (by CH) in the middle slice and copied to all slices of the *LMS IDEAL* PDFF maps to calculate PDFF statistics. The same ROIs were used to compute statistics from the Reference PDFF maps. Linear regression was computed for all PDFF statistics against the expected oil/water concentrations.

### Comparison of PDFF and T2* methods using UK Biobank data

Single slice abdominal MR images were acquired from the UK Biobank cohort at the UK Biobank Imaging Centre in Stockport, using a Siemens 1.5T MAGNETOM Aera. In a subset of the participants, (N = 179), two different imaging protocols were used to calculate PDFF and T2*, as shown in Table 1. Protocol1 data were used to calculate PDFF and T2* maps using *LMS IDEAL* (as described above). Protocol 2 data were used to calculate PDFF maps using the 3-point Dixon method [11] and T2* maps using a standard T2* decay-curve fitting method to measure the temporal relaxation rate of signal at each voxel (LMS Dixon and LMS T2* respectively). For quantification of each PDFF and T2* map, 3 circular ROIs of 15 mm diameter were placed manually on each map (by CH), within the right lobe of the liver, and carefully avoiding vessels and image artefacts. Mean values were calculated from the ROI pixels and compared between the two techniques for both PDFF and T2*. The UK Biobank has approval from the North West Multi-Centre Research Ethics Committee (MREC), and obtained written informed consent from all participants prior to the study.

**Table 1:** Image acquisition protocols used to calculate PDFF and T2* in UK Biobank cohort.

Parameter	Protocol1	Protocol2
PDFF Maps calc. using:	LMS IDEAL	LMS Dixon
T2* Maps calc. using:		LMS T2*
FOV (cm^2^)	44x40	40x40
Matrix	128x116	160x160
Voxel size (mm^2^)	1.7x1.7	2.5x2.5
Slice thickness (mm)	10	6
Flip angle (°)	5	20
TR (ms)	14	27
Pixel bandwidth (Hz/px)	1565	710
Number of averages	6	2
Number of echoes	6	10
First TE (ms)	1.2	2.38
Echo spacing (ms)	2	2.38
Breath-hold duration (s)	9.7 (1.6 / measure)	8.7

## Results

### PDFF in publicly available phantom data

**Fig 2:**
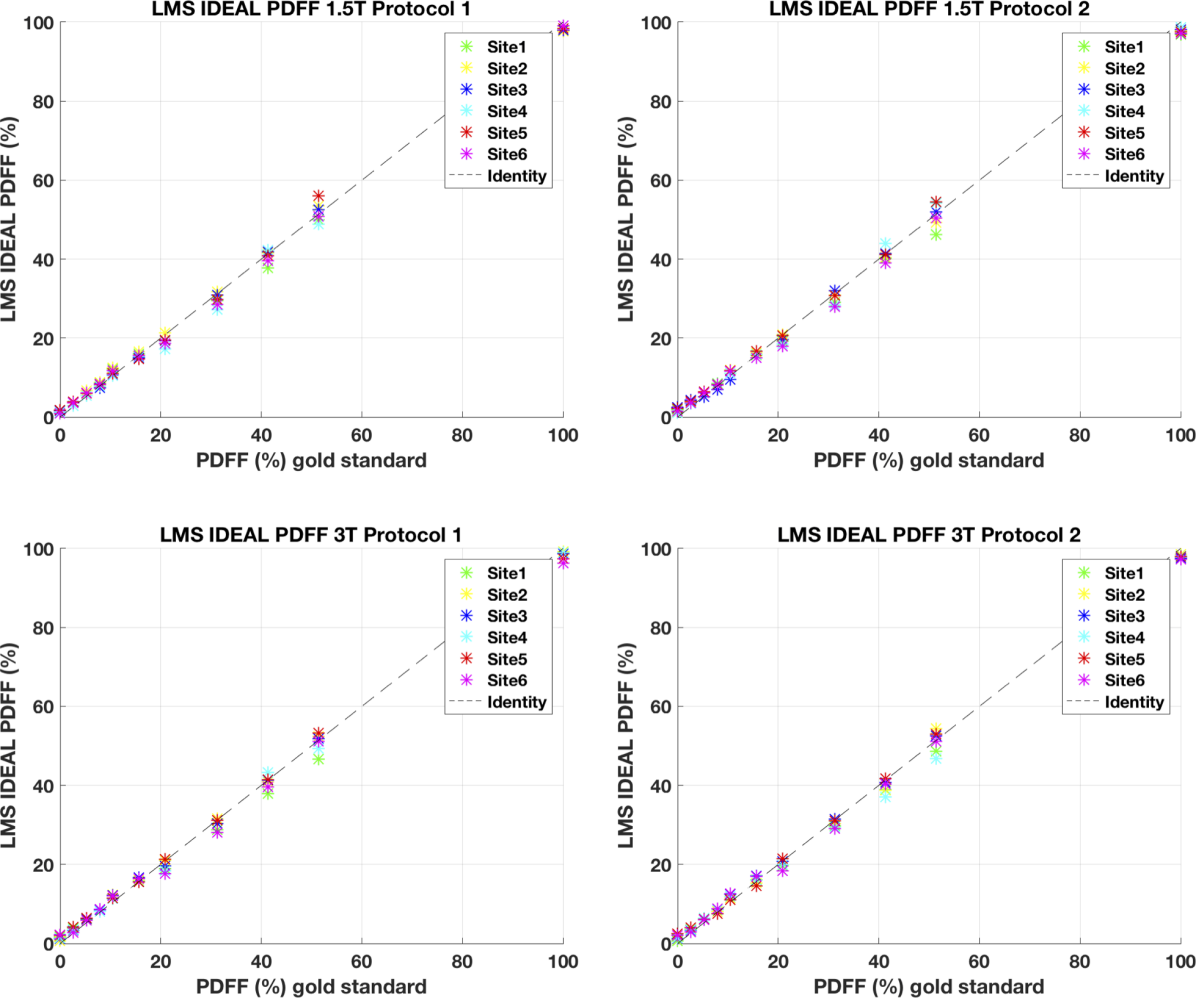
*LMS IDEAL* PDFF values versus gold standard values.

The *LMS IDEAL* PDFF values are plotted in Fig 2 against expected oil/water concentrations; Table 2 presents linear regressions with 95% confidence intervals. The linear regression results are in excellent agreement between *LMS IDEAL* PDFF and Reference PDFF, with a small reduction in either the r^2^ or increase in deviation of slope from 1, or intercept from 0 for *LMS IDEAL* PDFF. For example, for *LMS IDEAL* PDFF and Reference PDFF respectively, mean r^2^ = 0.998 and 0.999; mean slope = 0.970 and 0.995; mean absolute intercept = 0.72, and 0.26. From the plotted results in Fig 2, the small deviations appear to arise from the lowest PDFF value at the oil/water concentration of 0% and the one between 51.4%.

**Table 2:** Comparison of PDFF values using *LMS IDEAL* and Reference PDFF from Hernando et al., 2016.

	**LMS IDEAL PDFF 1.5T** **Protocol 1**			**Reference PDFF 1.5T** **Protocol 1**		
**Site**	***R***^***2***^	***Slope*, *[95% CI]***	***Intercept*, *[95% CI]***	***R***^***2***^	***Slope*, *[95% CI]***	***Intercept*, *[95% CI]***
**1**	0.998	0.96, [0.93 0.99]	0.72, [-0.54 1.99]	0.999	1.00, [0.97 1.03]	0.24, [-0.85 1.32]
**2**	0.999	0.97, [0.95 0.99]	1.48, [0.67 2.29]	1.000	1.02, [1.01 1.04]	0.69, [0.08 1.31]
**3**	0.999	0.98, [0.95 1.00]	0.58, [-0.40 1.56]	0.999	1.03, [1.01 1.05]	-0.43, [-1.26 0.40]
**4**	0.997	0.98, [0.93 1.02]	-0.28, [-1.90 1.35]	0.996	1.00, [0.95 1.04]	-0.57, [-2.28 1.14]
**5**	0.996	0.99, [0.94 1.03]	0.61, [-1.16 2.37]	0.999	1.00, [0.98 1.03]	0.40, [-0.58 1.38]
**6**	0.998	0.98, [0.95 1.01]	0.25, [-0.94 1.45]	0.998	0.99, [0.96 1.03]	-0.05, [-1.36 1.25]
	**LMS IDEAL PDFF 1.5T** **Protocol 2**			**Reference PDFF 1.5T** **Protocol 2**		
**Site**	***R***^***2***^	***Slope*, *[95% CI]***	***Intercept*, *[95% CI]***	***R***^***2***^	***Slope*, *[95% CI]***	***Intercept*, *[95% CI]***
**1**	0.998	0.94, [0.91 0.98]	0.84, [-0.48 2.17]	0.998	1.02, [0.99 1.05]	0.11, [-1.10 1.33]
**2**	1.000	0.96, [0.95 0.97]	1.10, [0.58 1.62]	0.999	1.02, [1.00 1.04]	0.83, [0.08 1.57]
**3**	0.998	0.98, [0.95 1.01	0.50, [-0.68 1.68]	1.000	1.01, [0.99 1.02]	-0.83, [-1.47–0.19]
**4**	0.996	0.99, [0.95 1.04]	0.48, [-1.29 2.24]	0.998	0.97, [0.94 1.01]	0.06, [-1.16 1.29]
**5**	0.998	0.97, [0.94 1.00]	1.36, [0.20 2.52]	1.000	0.98, [0.97 0.99]	0.80, [0.37 1.23]
**6**	0.998	0.96, [0.92 0.99]	0.31, [-0.99 1.61]	0.995	1.0, [0.96 1.06]	-0.67, [-2.66 1.31]
	**LMS IDEAL PDFF 3T** **Protocol 1**			**Reference PDFF 3T** **Protocol 1**		
**Site**	***R***^***2***^	***Slope*, *[95% CI]***	***Intercept*, *[95% CI]***	***R***^***2***^	***Slope*, *[95% CI]***	***Intercept*, *[95% CI]***
**1**	0.996	0.96, [0.91 1.00]	0.41, [-1.30 2.11]	0.999	1.00, [0.97 1.03]	0.02, [-1.10 1.14]
**2**	1.000	0.98, [0.97 1.00]	0.95, [0.42 1.48]	0.999	1.01, [0.99 1.03]	0.81, [-0.00 1.62]
**3**	0.999	0.98, [0.96 1.00]	0.86, [0.11 1.62]	0.999	1.01, [0.99 1.03]	0.23, [-0.67 1.12]
**4**	0.997	0.98, [0.94 1.02]	0.25, [-1.28 1.77]	0.998	0.97, [0.94 1.01]	0.30, [-0.92 1.53]
**5**	0.999	0.97, [0.95 0.99]	1.40, [0.57 2.23]	1.000	1.00, [0.99 1.01]	0.79, [0.31 1.27]
**6**	0.997	0.95, [0.91 0.99]	0.71, [-0.72 2.14]	0.997	0.98, [0.94 1.02]	-0.19, [-1.63 1.24]
	**LMS IDEAL PDFF 3T** **Protocol 2**			**Reference PDFF 3T** **Protocol 2**		
**Site**	***R***^***2***^	***Slope*, *[95% CI]***	***Intercept*, *[95% CI]***	***R***^***2***^	***Slope*, *[95% CI]***	***Intercept*, *[95% CI]***
**1**	0.999	0.97, [0.94 0.99]	0.40, [-0.57 1.38]	0.999	0.98, [0.96 1.00]	0.42, [-0.35 1.20]
**2**	0.998	0.98, [0.95 1.02]	0.51, [-0.88 1.90]	1.000	0.98, [0.97 0.99]	0.43, [-0.12 0.98]
3	0.999	0.97, [0.95 0.99]	1.36, [0.65 2.07]	0.999	0.97, [0.95 1.00]	1.06, [0.11 2.01]
**4**	0.997	0.95, [0.91 0.99]	0.59, [-0.98 2.15]	0.999	0.96, [0.94 0.98]	0.87, [0.12 1.62]
**5**	0.999	0.98, [0.95 1.00]	1.00, [-0.03 2.02]	0.999	0.98, [0.97 1.00]	0.73, [0.05 1.41]
**6**	0.998	0.96, [0.93 0.99]	0.95, [-0.33 2.22]	0.997	0.97, [0.93 1.01]	0.28, [-1.19 1.76]

### Comparison of PDFF and T2* methods using UK Biobank data

**Fig 3:**
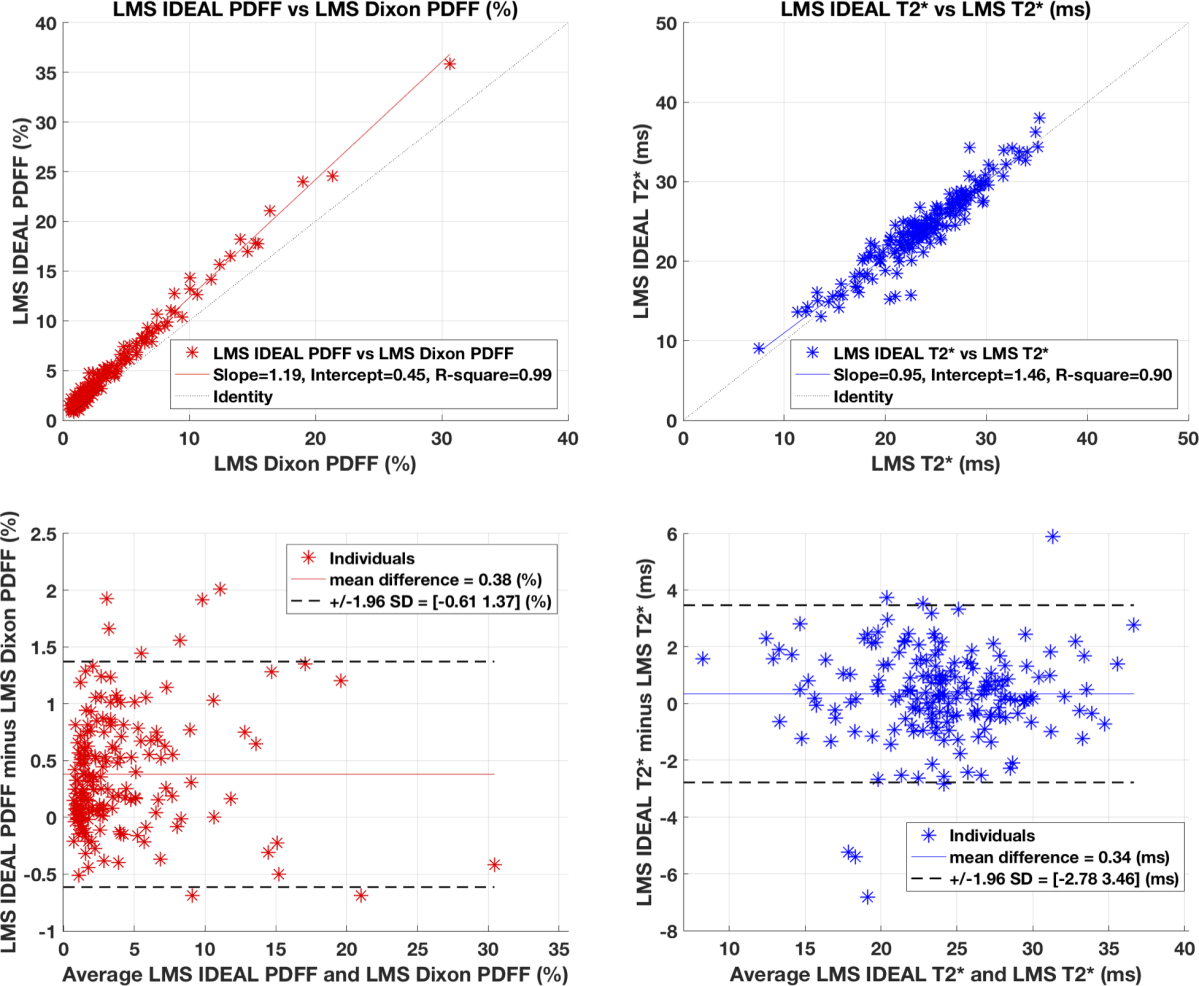
Comparison of PDFF and T2* methods using UK Biobank data.

The comparison between the two measures of PDFF are shown in Fig 3, top left. They show excellent correlation between the two protocols (r^2^ = 0.99), with regression slopes and intercepts = 1.19 and 0.45 respectively. *LMS IDEAL* PDFF is consistently higher than LMS Dixon PDFF, and is a function of PDFF: see the Discussion for an explanation of this. However, after correcting for the systematic difference using the regression slope, the Bland-Altman plot (Fig 3, bottom left) shows mean difference = 0.38% and 95% limits of agreement = [-0.61 1.37] %.

The comparison between the two measures of T2* are shown in Fig 3, top right. The results show a correlation r^2^ = 0.9, and regression slopes and intercepts = 0.95, 1.46 respectively. The T2* values are in excellent agreement as demonstrated in the Bland-Altman plot (Fig 3, bottom right) with mean difference = 0.34 ms, and 95% limits of agreement = [-2.78 3.46] ms. Furthermore, there was no statistical evidence for difference between the two protocols for T2* values using either of the fat models.

## Discussion

This study replicated results from previous work in a reproducible, controlled phantom experiment, and tested *LMS IDEAL* in a large number of human volunteers over a range of PDFF and T2* values. The phantom experiment suggests that *LMS IDEAL* is a standardized, confounder-corrected estimator of PDFF, which can be used across different vendors and field strengths. Limitations of phantom design and inter-acquisition variability, including effects of temperature and parameters of the acquisition protocols [24] are beyond the scope of this study. Sites 5 and 6 presented higher deviation in slope and intercept that can be attributed to differences in the approach to correct for bipolar readouts [24, 30].

The LMS Dixon and *LMS IDEAL* PDFF values measured from *in vivo* data show excellent correlation with systematic differences, which can be explained by differences in the acquisition protocol, and also by specific aspects of the model used to calculate PDFF values. The flip angle is the protocol parameter most likely to lead to PDFF differences (20° for Dixon versus 5° for IDEAL). This should decrease IDEAL PDFF relative to Dixon PDFF, the opposite of what is observed. The PDFF differences were then assessed using a single-peak fat model to calculate *LMS IDEAL* PDFF values, which is more similar to the LMS Dixon fat model. The resulting *LMS IDEAL* PDFF values were systematically lower than LMS DIXON PDFF values (results not shown). This may be attributable to the difference in flip angle (see e.g. (15)). The reduction in *LMS IDEAL* PDFF values using the single-peak fat model compared to the six-peak model can be attributed to a smaller contribution of the signal to the fat component ~1.5 (approx. 30% reduction). Summarising, the systematic difference between LMS Dixon and *LMS IDEAL* is attributable to different fat models, which leads to an under-estimation of PDFF for LMS Dixon that is then partly compensated by the increased flip angle for the LMS Dixon protocol. The *LMS IDEAL* T2* and LMS T2* results were in excellent agreement. Once all data has been acquired for the UK Biobank cohort, the *LMS IDEAL* acquisition protocol will have been used to acquire images in around 100K subjects. Further work is required to extend the phantom cross-vendor, cross field strength results to in-vivo data.

The results of this validation study demonstrates the potential for *LMS IDEAL* to be used as a standardised clinical tool for non-invasive quantification of biomarkers for liver diseases, which in turn enables applications in longitudinal clinical trials with multicenter participation.
